# Influence of hospital Accreditation on professional satisfaction of the nursing team: mixed method study^*^

**DOI:** 10.1590/1518-8345.2799.3109

**Published:** 2019-01-31

**Authors:** João Lucas Campos de Oliveira, Ana Maria Müller de Magalhães, Andrea Bernardes, Maria do Carmo Fernandez Lourenço Haddad, Lillian Daisy Gonçalves Wolff, Sonia Silva Marcon, Laura Misue Matsuda

**Affiliations:** 1 Universidade Federal de Mato Grosso, Faculdade de Enfermagem, Cuiabá, MT, Brazil.; 2 Universidade Federal do Rio Grande do Sul, Escola de Enfermagem, Porto Alegre, RS, Brazil.; 3 Universidade de São Paulo, Escola de Enfermagem de Ribeirão Preto, PAHO/WHO Collaborating Centre for Nursing Research Development, Ribeirão Preto, SP, Brazil.; 4 Universidade Estadual de Londrina, Departamento de Enfermagem, Londrina, PR, Brazil.; 5 Universidade Federal do Paraná, Departamento de Enfermagem, Curitiba, PR, Brazil.; 6 Universidade Estadual de Maringá, Departamento de Enfermagem, Maringá, PR, Brazil.

**Keywords:** Hospital Accreditation, Job Satisfaction, Quality Management, Nursing, Team, Personnel Management, Nursing Administration Research, Acreditação Hospitalar, Satisfação no Trabalho, Gestão da Qualidade, Equipe de Enfermagem, Administração de Recursos Humanos, Pesquisa em Administração de Enfermagem, Acreditación de Hospitales, Satisfacción en el Trabajo, Gestión de la Calidad, Grupo de Enfermería, Administración de Personal, Investigación en Administración de Enfermería

## Abstract

**Objective:**

to analyze the influence of Accreditation on the professional satisfaction of nursing workers.

**Method:**

multicentric, cross-sectional research, outlined by the sequential explanatory mixed method. In the first preponderant, quantitative step, the validated Brazilian version of the *Index of Work Satisfaction* was applied to a sample (n = 226) representative of nursing professionals from three hospitals. One hospital was private and certified by Accreditation; another hospital was private and non-certified; and another was public and non-certified. By connection, the second step (qualitative) complemented the quantitative analysis. In this step, interviews (n = 39) were carried out and summarized through the method of Discourse of the Collective Subject. Descriptive and inferential statistical analyses were applied to the quantitative data connected to the qualitative support, as well as a joint presentation of part of the information in a *joint display*.

**Results:**

workers of the certified hospital had a better overall job satisfaction score. There were more statistical associations among workers in private hospitals. The comparison of the three groups investigated in the two steps of the mixed study confirmed Accreditation as a positive factor for professional satisfaction. The public hospital excelled in relation to the certified hospital in terms of salary, job requirements and interaction.

**Conclusion:**

Accreditation positively influenced the professional satisfaction of the nursing teams investigated.

## RESUMO

**Objetivo:**

analisar a influência da Acreditação na satisfação profissional de trabalhadores de enfermagem.

**Método:**

pesquisa multicêntrica, transversal, delineada pelo método misto explanatório sequencial. Na primeira etapa preponderante, quantitativa, aplicou-se a versão brasileira validada do *Index of Work Satisfaction* a uma amostra (n=226) representativa de profissionais de enfermagem de três hospitais. Destes, um era privado e certificado pela Acreditação, e os demais, não certificados, sendo um público e outro privado. Por conexão, a segunda etapa (qualitativa) complementou a fase quantitativa por meio de entrevistas (n=39) que foram sumarizadas pelo Discurso do Sujeito Coletivo. Fez-se análise estatística descritiva e inferencial dos dados quantitativos conectados aos qualitativos de suporte, além de apresentação conjunta de parte das informações em *joint display*.

**Resultados:**

os trabalhadores do hospital certificado apresentaram melhor escore geral de satisfação profissional. Houve mais associações estatísticas entre os trabalhadores dos hospitais privados. A comparação dos três grupos investigados, nas duas fases do estudo misto, ratificou a Acreditação como fator positivo para a satisfação profissional. O hospital público sobressaiu-se em relação ao hospital certificado nas dimensões remuneração, requisitos do trabalho e interação.

**Conclusão:**

a Acreditação influenciou positivamente a satisfação profissional das equipes de enfermagem investigadas.

## RESUMEN

**Objetivo:**

analizar la influencia de la Acreditación en la satisfacción profesional de trabajadores de enfermería.

**Método:**

investigación multicéntrica, transversal, delineada por el método mixto explicativo secuencial. En la primera etapa preponderante, cuantitativa, se aplicó la versión brasileña validada del Index of Work Satisfaction a una muestra (n=226) representativa de profesionales de enfermería de tres hospitales. De ellos, uno era privado y certificado por la Acreditación, y los demás eran no certificados, siendo un público y otro privado. Por conexión, la segunda etapa (cualitativa) complementó la fase cuantitativa por medio de entrevistas (n=39) que fueron resumidas por el Discurso del Sujeto Colectivo. Se hizo análisis estadístico descriptivo e inferencial de los datos cuantitativos conectados a los cualitativos de soporte, además de presentación conjunta de parte de las informaciones en *joint display*.

**Resultados:**

los trabajadores del hospital certificado presentaron mejor puntaje general de satisfacción profesional. Hubo más asociaciones estadísticas entre los trabajadores de los hospitales privados. La comparación de los tres grupos investigados, en las dos fases del estudio mixto, ratificó la Acreditación como factor positivo para la satisfacción profesional. El hospital público sobresalió en relación al hospital certificado en las dimensiones remuneración, requisitos del trabajo e interacción.

**Conclusión:**

la Acreditación influenció positivamente la satisfacción profesional de los equipos de enfermería investigados.

## Introduction

Wrapped in a scenario full of adversity that is tied to the competitiveness of the globalized market, the health sector and health services should enable organizational survival through active, rational and, mainly, strategic management practices^1^. In this scope, based on the philosophy of quality management, evaluation is the aegis of the search for continuous improvements and indispensable practice for the rationalization and effectiveness of the work process^2^.

Due to the peculiarity of the *modus operandi* of the health area, evaluation can be a taboo. This differs from other service production branches such as aviation, hotel trade, and the financial/banking sector, which are nown to adopt evaluations in their daily work in a natural way. In spite of this, Accreditation has emerged throughout the world as a management system based, essentially, on third-party evaluation and demonstration of competence of the adherent organizations, with adaptations favorable to the unique reality of the health sector^3^.

The logic of Accreditation is mediated by the comparison of the institutional reality evaluated externally and periodically in the light of previously defined quality standards, determined in a clear and standardized way according to the accreditation methodology adhered by the country which, in the case of Brazil, is mostly represented by the National Accreditation Organization (NAO)^3^ ^-^ ^4^. This logic reaffirms that Accreditation is conducted through the establishment of criteria, standards and indicators to leverage the strategic vision of institutions, which are driven by external evaluation^2^.

Although the main focus of Acreditation is the clinical-healthcare dimension, because it has emerged in the light of health production, this quality management system tends to have a positive impact on services in several organizational spheres, such as changes in top management, cost administration, and business *marketing* ^3^ ^,^ ^5^. However, the real benefits of Accreditation, whether in the health care or administrative/strategic context, are still little known, both nationally and internationally and then studies are still needed to identify improvements attributable to this quality management system^5^ ^-^ ^8^.

Considering the impossibility of dissociating the nursing services from the quality of care provided, including in hospitals^9^, it is postulated that the knowledge *gap* on the benefits of Accreditation is related to the professional satisfaction of the nursing team because, in theory, the dynamics of people management needs to be improved through adherence of organizations to Accreditation^4^.

The professional satisfaction of the nursing team is an important indicator of the quality of organizational results, especially in the context of human resource management. This is understood as a complex and multidetermined phenomenon that converges to a behavioral state of the worker toward his work^10^. Therefore, knowing the professional satisfaction of the nursing team can direct a relevant and dual perspective of evaluation, namely: the professionals’ view about their work, and also the potential to impact the produced care^10^ ^-^ ^11^.

The fact that professional satisfaction is an interest towards the improvement of nursing services and that investigating the real benefits of Accreditation to the overall quality of health is sorely needed^2^ ^,^ ^5^ ^-^ ^8^ ^)^ makes research on this theme necessary, especially the multicenter studies, i.e. those coducted in different places.

Once the inclination to investigate the problem has been justified, this study was guided by the following questions: “Does Accreditation influence the professional satisfaction of nursing workers? If so, in what way?”. To answer these questions, the objective was to analyze the influence of Accreditation on the professional satisfaction of nursing workers.

## Method

Multicentric, cross-sectional research, outlined by the sequential explanatory mixed method. In this study design, the research is conducted in steps of the following approaches: first, the quantitative (QUAN) analysis, which is priority and has a greater weight, and, second, the qualitative (Qual) analysis, of lesser weight^12^.

In order to be confirmed as a sequential explanatory mixed study, the approaches/steps of the study need to be interrelated by a connection procedure in which the analysis of the data collected from the “QUAN” step leads to the collection of data in the “Qual” step^12^.

The study sites were all Intensive Care Units (ICUs) of three hospitals located in the country side of the state of Paraná, Brazil. The NAO *website* was initially searched for selection of sites. A general hospital located in Paraná with beds in Intensive Care Units (ICUs) granted the highest level (Accredited with Excellence) of certification by the accreditation methodology in question, and that had the greatest durability for expiration, was searched in the NAO website^4^. After selection of the Accredited Hospital (HA), which was private, two other general hospitals with intensive care beds were selected at most 150 kilometers distant from the AH and of the same size (medium size) of the AH.

Two other non-accredited hospitals were selected through the National Registry of Health Establishments. Of these, one was private (HB) and the other was public (HC). This procedure of differentiating the type of care between the two hospitals to be compared to the certified organization (HA) was intentional and aimed to increase the possibility and variability of inferences about the objects of study. In turn, the procedure of maximum distance between institutions was adopted to reduce variation (deriving from geographic/cultural realities) of labor issues that could influence the professional satisfaction, such as working hours and remuneration.

The study population consisted of nursing professionals working in the ICU of the three hospitals. With the total number of workers in the sectors (according to professional category and unit of work), provided by the management of the respective nursing services, a stratified representative sampling was calculated based on finite populations, with a coverage level of 95% and margin of error of 5%.

The sample was stratified in two ways in each of the hospitals: segregation of the workers between nurses and professionals without superior education, and the sample size was based on subjects working in the “Adult” ICU - ICU-A (general intensive care unit, coronary care unit, cardiologic care unit etc.) and ICU “Infant” - ICU-I (neonatal intensive care unit and pediatric intensive care unit). These procedures were performed to reach a good representativeness of the entire population of each of the sites investigated.

Once the sampling design was established, data from the first step (QUAN) were collected between December 2016 and March 2017, with a convenience approach, in all shifts and units of the three hospitals surveyed. In order to be included in the sample, the worker should meet the only inclusion criterion which was to work in the ICU for at least six months, according to the recommendation of a previous study on professional satisfaction^10^. The collection was carried out at each site until completing the sample stratified per professional categories and units of action, based on the application of part of the validated Brazilian version of the *Index of Work Satisfaction* (IWS)^10^.

The part of the IWS used in the study covered the sociodemographic and labor characterization of the nursing team, together with the entire “part B” of the second portion of the instrument, *composed of a Likert* type scale concerning the six domains/dimensions of job satisfaction^10^. These parts were enough to reach the proposed objective, which was not to determine job satisfaction, but rather the influence of Accreditation in this phenomenon studied according to domains.

The domains of the IWS are: *Remuneration*, *Professional Status*, *Autonomy*, *Organizational Norms*, *Job Requirements* and *Interaction*. The latter may or may not be unfolded into nurse-physician interaction and nurse-nurse interaction^5^ ^,^ ^10^. In this study, the option was not to make such differentiation. In these six dimensions, the scale is distributed in 44 evaluative items raging from 1 (total agreement) to 7 (total disagreement); the lower the score, the greater the agreement of the subject with the phrase, which was translated into greater/better professional satisfaction^10^.

Following the design of a mixed sequential explanatory study, after data collection of the first stage (QUAN), the points of interest were surveyed to be confirmed/deepened/contrasted in the second step (Qual), representing the interrelation of data by connection^12^. This was done through the descriptive statistical analysis of the quantitative data collected. In the descriptive analysis, the IWS domains were analyzed by the average score of their evaluative items, besides the use of amplitude (minimum and maximum) and dispersion (standard deviation) measures.

After the primary quantitative analysis, differences of positive and negative points in the dimensions of job satisfaction among the three hospitals were identified, that is, the results were heterogeneous. This confirmed the need to continue the mixed research based on all dimensions of the IWS in order to clarify the divergences noted and not to delve into any specific point. Then, data collection of the second step (Qual) of the study was carried out through interviews guided by all dimensions measured by the IWS^10^.

The participants of the second step of the study were nursing workers of all levels of education who were in the same sectors and institutions, and who participated in the primary quantitative phase. Data collection from the “Qual” step was carried out based on a convenience sample, in June 2017, in all shifts of work of the three hospitals. To do so, workers were first randomly approached and asked whether he/she had answered the IWS questionnaire in the previous stage (QUAN). If they had answered the instrument, they would be invited to accept informally the collection of qualitative data in the second step.

Qualitative data were collected through structured individual interviews respecting the dimensions of professional satisfaction under study^10^. To this end, the participants were asked to express their view on the six IWS domains using directive questions such as “*Tell me how you feel about your salary*”. The freedom of expression of each participant was respected, however, whenever necessary, the interview was (re)directed to the focus of interest. The collection took place until the moment the statements became repetitive, per IWS domain, in each hospital.

After data from the two steps were collected, the final analysis was performed. In this moment, the data of the “QUAN” step tabulated and already analyzed descriptively were subjected to inferential analysis in the *Statistical Package for the Social Sciences*, version 21 (SPSS-21).

First, the normality of the data of the IWS scale was checked by the *Kolmogorov-Smirnov* test. In case of normal distribution, the analysis continued with the *t-student* parametric test for independent samples to compare two groups, based on the “Accreditation factor” (HA x HB; HA x HC), and *one-way* ANOVA for comparisons of three groups. The statistical significance adopted in all inferential analyses was 5%, expressed as p-value and Confidence Interval between differences. The reliability (internal consistency) of the *IWS was tested and considered satisfactory when Cronbach’s alpha* was higher than 0.7^13^.

The data of the step “Qual” were transcribed verbatim in a digital medium. After that, the printed material was analyzed by the Discourse of the Collective Subject (DCS) to group the previously thematized data according to the previous connection to IWS domains and then presented in first-person speech, so that the testimonies, in each hospital, were unified according to the domains of professional satisfaction^14^. This means that the DCS was used as a technique for grouping the data that had already been directed to thematic organization in the collection according to the IWS dimensions, in order to converge to the sequential explanatory mixed methodology^12^.

In the presentation of the findings, the statements were edited educational standards but without changing the meaning in the DCS. Understanding each discourse as coming from a colectivity^14^, the agglutinated statements were identified only by the reference hospital (HA, HB and HC). Furthermore, the averages of the IWS domains were re-presented in order to corroborate or confront the content of each DCS and present the results of the different approaches in the mixed research jointly, as recommended by contemporary scholars in mixed methods such as “*joint display*”^15^.

All ethical precepts governing human research were respected, including the use of the Informed Consent Term in the two steps of data collection. Thus, the study was submitted and approved by the institutionalized Ethics Committee and is registered nationally with CAAE: 58571216.4.0000.0104.

## Results

The first step (QUAN) of the study had a representative sample of 226 nursing workers from the three hospitals. Of these, 82 (36.2%) belonged to HA; 59 (26.2%) to HB and 85 (37.6%) to HC. In all hospitals, the proportion (n = 72, 87.8%, n = 52, 88.1%, and n = 75, 82.2%) of women prevailed.

Of the total, 56 (24.8%) professionals were nurses and the others (75.2%) were nursing technicians (n = 165) or nursing assistants (n = 5). Among the nurses, 41 (73.2%) reported to work directly with provision of care and 15 (26.8%) reported to work with both care provision and management. Regarding the sector (adult or child), 125 (55.4%) were in adult ICU and 101 (44.6%) in child ICU.

The mean age of the workers, per hospital, was HA = 36 (± 8.6); HB = 30 (± 8.4); and HC = 39 (± 8). The average monthly income of professionals in Reais (R$), was: HA = 2,158 (± 957); HB = 1738 (± 723); and HC = 5.030 (± 2197). In turn, the average time working in the unit, in years, was as follows: HA = 6.3 (± 4.7); HB = 3.4 (± 3.7); and HC = 7.4 (± 5.4).

Table 1 shows the descriptive results and reliability of the IWS domains, per hospital (HA, HB and HC), as well as general data of the sample.

**Table 1:** Descriptive data of the domains of professional satisfaction and the reliability test among nursing workers, per hospital (n = 226). Paraná, Brazil, 2016-2017

**Hospital**		**Minimum**	**Maximum**	**Mean**	SD*	**Reliability** ^**†**^
**A**	**Remuneration**	**1**	**6**	**3.44**	**1.048**	**0.716**
	*Professional Status*	**1**	**5**	**2.58**	**0.887**	**0.556**
	**Autonomy**	**1**	**7**	**3.60**	**1.067**	**0.702**
	**Organizational Standards**	**1**	**6**	**3.33**	**0.930**	**0.621**
	**Job Requirements**	**1**	**7**	**4.12**	**1.073**	**0.601**
	**Interaction**	**1**	**6**	**3.43**	**0.934**	**0.659**
	**Total**	**2**	**6**	**3.34**	**0.634**	**0.851**
**B**	**Remuneration**	**2**	**6**	**4.33**	**0.907**	**0.480**
	*Professional Status*	**1**	**4**	**2.66**	**0.689**	**0.094**
	**Autonomy**	**2**	**6**	**4.00**	**0.868**	**0.481**
	**Organizational Standards**	**1**	**6**	**3.71**	**0.870**	**0.547**
	**Job Requirements**	**1**	**7**	**4.38**	**1.05**	**0.604**
	**Interaction**	**1**	**6**	**3.65**	**0.871**	**0.574**
	**Total**	**2**	**5**	**3.76**	**0.597**	**0.808**
**C**	**Remuneration**	**1**	**6**	**3.04**	**0.882**	**0.589**
	*Professional Status*	**1**	**6**	**2.60**	**0.834**	**0.546**
	**Autonomy**	**2**	**6**	**3.70**	**0.952**	**0.671**
	**Organizational Standards**	**2**	**5**	**3.78**	**0.719**	**0.363**
	**Job Requirements**	**2**	**6**	**4.08**	**0.939**	**0.524**
	**Interaction**	**1**	**6**	**3.38**	**0.943**	**0.724**
	**Total**	**2**	**5**	**3.43**	**0.608**	**0.848**
**Overall**		**1**	**7**	**3.50**	**0.631**	**0.844**

*SD - Standard Deviation; ^†^Reliability (Measured by *Cronbach’s Alpha*).

Table 2 shows inferential data in the presentation of the results among professionals of the accredited hospital and the non-accredited private hospital.

**Table 2:** Comparison of professional satisfaction domains of nursing workers from the accredited hospital (HA) and the non-accredited private hospital (HB) (n = 141). Paraná, Brazil, 2016-2017

**Domain**	**Hospital**	**Mean**	SD*	**95% CI** ^**†**^	**p-value** ^**‡**^
**Remuneration**	**A**	**3.44**	**1.048**	**[-1.22 - -0.548]**	**0.000**
	**B**	**4.33**	**0.907**		
**Professional status**	**A**	**2.58**	**0.887**	**[-0.361 - 0.203]**	**0.567**
	**B**	**2.66**	**0.689**		
**Autonomy**	**A**	**3.60**	**1.067**	**[-0.754 - -0.045]**	**0.027**
	**B**	**4.00**	**0.868**		
**Organizational Standards**	**A**	**3.33**	**0.930**	**[-0.703 - -0.063]**	**0.019**
	**B**	**3.71**	**0.870**		
**Job Requirements**	**A**	**4.12**	**1.073**	**[-0.630 - 0.108]**	**0.164**
	**B**	**4.38**	**1.053**		
**Interaction**	**A**	**3.43**	**0.934**	**[-0.548 - 0.102]**	**0.177**
	**B**	**3.65**	**0.871**		
**Overall**	**A**	**3.34**	**0.634**	**[-0.680 - -0.165]**	**0.002**
	**B**	**3.76**	**0.597**		

*SD - Standard Deviation; ^†^95% CI (Confidence Interval between differences); ^‡^p-value (*t-student* test).

Table 3 shows data of the association of professional satisfaction domains, considering the presence/absence of the accreditation factor in two groups, one of which is represented by professionals of the non-accredited public hospital (HC).

**Table 3:** Comparison of professional satisfaction domains of nursing workers from the accredited hospital (HA) and the non-accredited public hospital (HC) (n = 167). Paraná, Brazil, 2016-2017

**Domain**	**Hospital**	**Mean**	SD*	**95% CI** ^**†**^	**p-value** ^**‡**^
**Remuneration**	**A**	**3.44**	**1.048**	**[0.095 - 0.697]**	**0.010**
	**C**	**3.04**	**0.882**		
**Professional status**	**A**	**2.58**	**0.887**	**[-0.298 - 0.252]**	**0.871**
	**C**	**2.60**	**0.834**		
**Autonomy**	**A**	**3.60**	**1.067**	**[-0.433 - 0.223]**	**0.528**
	**C**	**3.70**	**0.952**		
**Organizational Standards**	**A**	**3.33**	**0.930**	**[-0.715 - 0.186]**	**0.001**
	**C**	**3.78**	**0.719**		
**Job Requirements**	**A**	**4.12**	**1.073**	**[-0.277 - 0.355]**	**0.808**
	**C**	**4.08**	**0.939**		
**Interaction**	**A**	**3.43**	**0.934**	**[-0.254 - 0.347]**	**0.760**
	**C**	**3.38**	**0.943**		
**Overall**	**A**	**3.34**	**0.634**	**[-0.331 - 0.139]**	**0.419**
	**C**	**3.43**	**0.608**		

*SD - Standard Deviation; ^†^95% CI (Confidence Interval between differences); ^‡^p-value (*t-student* test).

Table 4 shows the data of the last quantitative analysis, which consists of the statistical association between the three groups of workers of the hospitals.

**Table 4:** Association of domains of professional satisfaction among nursing workers of the three hospitals (n = 226). Paraná, Brazil, 2016-2017

**Dimension**	**Hospital**	**Mean**	SD*	**F** ^**†**^	**p-value** ^**‡**^
**Remuneration**	**A**	**3.44**	**1.048**	**31.594**	**0.000**
	**B**	**4.33**	**0.907**		
	**C**	**3.04**	**0.882**		
**Professional status**	**A**	**2.58**	**0.887**	**0.156**	**0.855**
	**B**	**2.66**	**0.689**		
	**C**	**2.60**	**0.834**		
**Autonomy**	**A**	**3.60**	**1.067**	**2.683**	**0.071**
	**B**	**4.00**	**0.868**		
	**C**	**3.70**	**0.952**		
**Organizational Standards**	**A**	**3.33**	**0.930**	**6.037**	**0.003**
	**B**	**3.71**	**0.870**		
	**C**	**3.78**	**0.719**		
**Job Requirements**	**A**	**4.12**	**1.073**	**1.626**	**0.199**
	**B**	**4.38**	**1.053**		
	**C**	**4.08**	**0.939**		
**Interaction**	**A**	**3.43**	**0.934**	**1.503**	**0.225**
	**B**	**3.65**	**0.871**		
	**C**	**3.38**	**0.943**		
**Overall**	**A**	**3.34**	**0.634**	**6.121**	**0.003**
	**B**	**3.76**	**0.597**		
	**C**	**3.43**	**0.608**		

*SD - Standard Deviation; ^†^F (*F-ratio* of comparison of variances); ^‡^p-value (*one-way* Anova).

**Figure 1:**
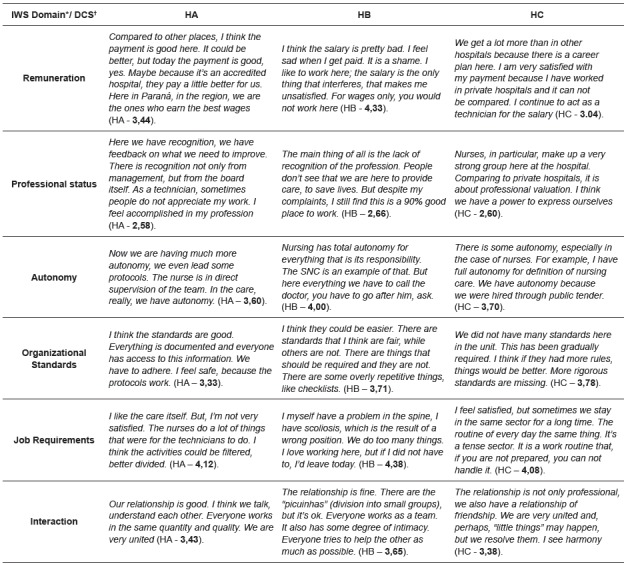
Summarized statements in Discourses of the Collective Subject (DCS) guided by the domains of professional satisfaction and their respective means among nursing professionals, per hospital. Paraná, Brazil, 2017

The second step (Qual) of the study involved 39 interviews with participants of the first step (QUAN) distributed in the order of hospitals (HA, HB, and HC), respectively, in 14, 10 and 15 interviews. Respecting the sequence of the mixed explanatory study, the DCS guided by the domains and their means of IWS is presented, in *joint display*, according to hospital (Figure 1).

* IWS - Index of Work Satisfaction; † DCS - Discourses of the Collective Subject.

## Discussion

It is noticeable that the sample was young in all hospitals, but the public institution had the highest average age and also time of work in the units, which undoubtedly is linked to the dynamics of recruiting staff that is mostly through public tender, creating greater employment stability. Among the private institutions, professionals of the accredited hospital had a higher average age and practically double of the time of work in the sector in comparison with HB. This data can mean an improvement in the management of people in the accredited institution through retention of talents, which is pointed out as an indicator of organizational quality at the heart of personnel management^16^.

A fact that is out of the objective of the research but that called attention in a critical reflection of the findings was the greater proportion of nurses who reported to be dedicated “only” to care provision in their units. This may be a product of the service organization itself through a chart that includes the average management in the ICUs as the leadership of the “assistance” nurses. However, not to recognize themselves as care managers - regardless of the position occupied - weakens the professional identity of nurses and the consolidation of nursing as a science.

It is important that the recognition or acceptance of nurses as care managers be debated since their professional training because, although nursing management and nursing care are complementary and inseparable, there is a dichotomy between them since undergraduate training and this can negatively affect the future professional identity and, perhaps, job satisfaction^17^. In contrast, despite the relevance of taking management to the work scope of nursing in any sphere of work, nurses occupying strategic-administrative positions were less satisfied than their peers who dedicate their attention to “assistance” positions^18^.

In relation to the average salary found in the three research sites, the positive contrast between the public institution and, secondly, the accredited organization, which paid the highest salaries, was evident. This raw/isolated data is confirmed by assessments around this field of job satisfaction, either through quantitative or qualitative analysis. That said, it is important to recognize the positive statistical significance in the comparisons of two groups, and it can be inferred that remuneration was a domain influenced by Accreditation in the analysis among private institutions, but in the comparison with a non-accredited public hospital, the results were better for the latter.

Remuneration is not the only factor that influences professional satisfaction, but it is relevant for any worker, because favorable conditions to the quality of life demand financial resources^10^ ^-^ ^11^ ^,^ ^18^. In this respect, it is praiseworthy that the accredited hospital, although private, presented a higher average payment of personnel than the non-accredited private institution. This may be directed to a less mercantile view of work and greater appreciation of the human capital, producer of care, respecting the external needs to the work.

The evident difference between the remuneration in the three hospitals is an aspect that deserves attention, especially the lower value paid in the HB to nursing workers that may be incompatible with the quality of life of these professionals. It is postulated that although the issue of remuneration does not legitimize the valorization of work by itself, needs to be continually rethought by the leaderships and class entities of the profession, also with respect to the militancy in favor of the minimum wage paid to these professionals.

Although remuneration is an important aspect in the perception of job satisfaction, the study confirms that it is not isolated, because despite higher payment, the nursing staff of the public hospital, in general, did not seem to be the most satisfied. In the general perception of the sample of the studied sites, the average scores of the employees of the accredited hospital were better.

The non-accredited private institution obtained the worst scores, both in the general analysis and also in the IWS domains when compared to HA. This again points to Acreditation as a possible intermediary factor of better working conditions in private companies. This was confirmed by the more positive evaluation of professional *status*, whose domain is translated by the IWS as social and organizational recognition of work, which, indeed, was better at the accredited hospital, with quality seal^10^.

This highlights the previous assumption found in the Handbook of Health Service Organizations of the NAO, in the Management and Leadership section and People Management subsection. These express, in the requirements of the quality standard of certification level 2, the need of the organization to analyze and promote improvement actions for programs related to the quality of life and health of employees, based on actions for job satisfaction^4^.

At the third level of certification (Accreditation with Excellence), as in the case of HA, the institution needs to demonstrate excellence in management through rational strategies over measured results in this and other evaluation perspectives, highlighting the organization’s commitment to its human capital^4^ ^-^ ^5^.

It can be seen that the logic of rationalization of health work mediated by Accreditation can be a factor that contributes to professional satisfaction of the nursing team. This is ratified by the data analyzed from the three groups of workers, in which the team working in the accredited hospital had the higher total score. This data alone can be considered relevant and even innovative for the knowledge of the management system investigated. Even with some similar scores, Accreditation was a factor that defined statistical significance in several evaluative dimensions, either in paired or condensed comparison in the three groups.

In the overall analysis of the mixed study, the DCS of HA corroborates the positive view of satisfaction of this group of workers, especially when contrasted with the DCS of HB. It is therefore believed that the global interpretation that Acreditation positively interfered with the phenomenon of work satisfaction is reinforced, although limited to the researched institutions.

The overall scores of all hospitals were close to the median of the IWS, that is, they tended to neutrality in the evaluation of the scale. This indicates that the variability of items and the evaluative dimensions influence the determination of general satisfaction, and reaffirms it as a multidetermined phenomenon. Although the variability of items is a reality in the application of the IWS, the constructs of the instrument in each hospital and, also, in the general measurement of the total sample proved to be reliable in their measurement according to the *Cronbach’s Alpha*, which in a way strengthens the inferences emanating in the present study.

The findings related to the IWS domains allowed a clearer understanding of what was intended to be studied, identifying Accreditation as an interest factor, a mediator of better job satisfaction. This is especially so in the dimensions *professional status* and *organizational norms*, because these were evaluated more positively by the employees of the accredited hospital, either in comparison with the public or the private institution not accredited and with quality seal.

Even more clearly, in the dimension organizational norms, the testimonies reinforce the better job satisfaction in the hospital that adheres to accreditation. This was also ratified by the greater difference found in the comparison of average scores in the field evaluated by the IWS, a fact that placed the mixed study as an approach that promotes greater deepening and understanding of the investigated phenomenon.

As a management system based on the rational logic of the basic principles of management, but adapted to the unique reality of the health area, Accreditation tends to be clearly standardized, even because its well-defined principles and methods are based on full compliance with standards (i.e., the understanding of assessment for certification is based on the perspective of “all or nothing” in the pursuit of compliance with standards)^3^ ^-^ ^4^.

In the Accreditation process, it is expected that management practices, especially in hospitals - where the pace of work is troubled and the technological density associated with care is high - that adhere to this quality management system be strictly rational, punctual and strategic, resulting in a formal apparatus of well-mapped rules, protocols, routines and work processes^5^.

When organizational norms are placed in check, it is reminiscent of the empirical but socially known conception that nursing workers may be “plastered” to caring actions or otherwise oppressed by a robust organizational apparatus. In the context of Accreditation, it must be acknowledged that the system is already regarded as a promoter of tension due to excessive collection of results and also for causing greater stress among Brazilian nurses, which may or may not be linked to the excessive standardization of work^19^ ^-^ ^20^.

The findings of this study suggest that the professionals in the institution accredited with excellence attributed satisfaction to the “imposed” organizational norms. In the qualitative part of the research, such norms were related to markers of standardized conducts and better job security, for the adoption of protocols and well defined conducts. This is in line with a research conducted with 220 Korean nurses, in which Accreditation from the *Joint Commission International* was envisaged as an intermediary of development and better professional performance^21^.

It is noteworthy that this research happened in two non-accredited institutions and in a hospital accredited with the highest level of the Brazilian methodology. Adding the results that reflect the satisfaction with the organizational norms to the data of characterization of the sample leads to the reflection of the fact that, due to the durability in the employment and the possible solid adherence to the principles of excellence in management, the workers of HA, in fact, perceive labor standards as facilitating aspects of the labor process and not as a bureaucratic contribution. This was seen in the DCS of the field in question, referenced in HB, or the “lack” of standards, demonstrated in the DCS of HC.

The findings of a study carried out with 901 health professionals from Saudi Arabia corroborate with the results of this study, who referred to Accreditation as responsible for direct improvements in the work process, in face of their normative requirements^6^. On the other hand, another investigation carried out with 1312 nurses from Iran testifies that, despite bringing robustness to the work process, the impact of Accreditation on the quality of concrete results can not yet be taken as absolute truth^22^. 

Regarding Accreditation as the only factor interfering with job satisfaction is premature and counterproductive, since the work dynamics in any organization hold variables that influence job satisfaction and other indicators, far beyond the management system or its absence. This is in line with the frankness of the fact that isolating the “Accreditation factor” is a challenge. Possibly, this is the major limitation of this research, besides being restricted to hospitals in the state of Paraná.

In spite of the above, the study brings interesting answers and different concreteness by the methodological contribution. Especially in the Brazilian context, where the scarcity of knowledge about the benefits attributable to Accreditation is still real, it is believed that the research advances solidly to what is known about this management system. It also contributes to nursing science, in especially in the area of management of human resources, for reaffirming - in the light of quality management - professional satisfaction as an indicator of interest in the evaluation of services.

## Conclusion

It is concluded that Accreditation positively influenced the professional satisfaction of nursing workers, since significant statistical associations were found both in the peer evaluations and in the analysis among the three groups. In the comparison between private institutions, all domains were evaluated more positively by the employees of the accredited hospital. The qualitative secondary dimension of the mixed study ratified the favorable perspective of Accreditation, also reinforcing the greatest comparative difference between non-public institutions.

It should be emphasized that the isolated assignment of the best satisfaction in nursing work in relation to Accreditation is premature. Further studies are needed, as for example longitudinal research, with an approach encompassing different levels of certification and, mainly, direct results on care linked or not to job satisfaction. To this end, mixed research is undoubtedly an alternative to be elected.
